# Is high-quality robotic surgery a solo performance by the console surgeon?

**DOI:** 10.1093/gastro/goag102

**Published:** 2026-09-16

**Authors:** Jun Lu, Junwei Zou, Youdong Liu, Fenglin Liu

**Affiliations:** Department of Gastric Surgery, Fudan University Shanghai Cancer Center, Shanghai, P. R. China; Department of Medical Oncology, Shanghai Medical College, Fudan University, Shanghai, P. R. China; Department of Gastrointestinal Surgery, The Second Affiliated Hospital of Wannan Medical University, Wuhu, Anhui, P. R. China; Department of Gastric Surgery, Fudan University Shanghai Cancer Center, Shanghai, P. R. China; Department of Medical Oncology, Shanghai Medical College, Fudan University, Shanghai, P. R. China; Department of Gastric Surgery, Fudan University Shanghai Cancer Center, Shanghai, P. R. China; Department of Medical Oncology, Shanghai Medical College, Fudan University, Shanghai, P. R. China

Kim *et al.* [1] and Ferzli *et al.* [2] recently emphasized that the rapid expansion of robotic surgery should not come at the expense of laparoscopic proficiency in surgical training. We agree with this point. In addition, we believe that another key message should not be overlooked: high-quality robotic surgery is not a solo performance by the console surgeon, but requires continuous collaboration with a highly skilled bedside assistant possessing advanced laparoscopic skills. The bedside assistant undertakes critical tasks, including suction, exposure, retraction, vascular clipping, tissue transection and stapling, and management of intraoperative emergencies. The bedside assistant’s technical proficiency directly affects the clarity of the operative field, the pace of the procedure, and patient safety [3]. From this perspective, robotic surgery has not eliminated the need for laparoscopic skills; rather, it has reassigned some essential laparoscopic skills to the assistant.

Robotic radical gastrectomy provides a representative example, as the assistant’s appropriate use of the suction device most clearly illustrates this ‘redistribution of skills’. Suction is not merely used to remove fluid; it is an important means of establishing and maintaining the operative field. Robotic systems provide greater magnification than conventional laparoscopy; consequently, bleeding can more rapidly and significantly compromise visualization compared with conventional laparoscopic surgery. For example, during suprapancreatic lymph node dissection (at the root of the left gastric vessels, or around the splenic vessels), oozing or active bleeding may rapidly obscure the operative field with accumulated blood, compromising the surgeon’s working space. The assistant must help the console surgeon identify the bleeding point rapidly and accurately, select an appropriate suction position, angle, and force according to the direction of bleeding and the relevant anatomical planes without injuring surrounding tissues, and switch rapidly among suction, targeted compression, and tissue retraction to facilitate prompt hemostasis. Suction that is too superficial cannot effectively remove accumulated blood, whereas suction that is too deep may draw tissue into the suction tip, place traction on vessels, and obstruct the endoscopic view. Precise suctioning is therefore itself a laparoscopic skill requiring spatial judgment and hand-eye coordination, rather than a simple ancillary task [4].

The importance of safe and precise placement of hem-o-lok clips likewise should not be underestimated. Robotic instruments can accomplish meticulous dissection; however, in many gastric cancer surgical procedures, vascular clips are still applied by the assistant through the assistant port [5]. Safe clip application requires more than merely placing a clip across the vessel. It also requires confirmation that the vessel has been adequately dissected free, assessment of the relationship between the clip applier and the course of the vessel, and control of clip orientation, vascular stump length, and the distance between the clip and surrounding tissues, so as to avoid oblique clip placement, incomplete closure, or inadvertent clipping of adjacent tissues. At the same time, the assistant must plan the instrument insertion trajectory, avoid the endoscope and robotic arms, and complete clip application without obstructing the console surgeon’s view or disrupting the rhythm of dissection. All of these details depend on sound laparoscopic instrument-control skills and anticipation of the next operative step.

For gastrointestinal reconstruction, high-quality robotic surgery places substantial demands on the assistant’s laparoscopic skills. In mainland China, robotic linear cutting staplers are not yet in routine use; therefore, transection and anastomosis of the stomach, esophagus, and bowel must still be performed by the assistant using a conventional laparoscopic linear cutting stapler through the assistant trocar port [6, 7]. The assistant must control the insertion direction of the stapler, cartridge position, extent of tissue transection, angle of closure, and timing of firing. Even if robotic staplers become more widely used in the future, they will primarily change who controls the device and cannot replace the operative preparation and assessment required before anastomosis. During anastomosis, the gastric remnant, esophagus, and jejunum must be appropriately retracted and flattened to achieve a torsion-free, low-tension anastomotic configuration with reliable blood supply [8]. Anastomotic quality is therefore determined not only by a single firing, but also by the assistant’s continuous assessment and control of tissue orientation, tension, and spatial relationships.

Furthermore, the surgeon and the bedside assistant do not operate under equivalent visual and operative conditions. The former typically has a magnified, immersive 3D view and wristed instruments, whereas the latter generally uses conventional non-wristed laparoscopic instruments while viewing a 2D monitor. The assistant must reconstruct spatial relationships from 2D cues, including tissue overlap, instrument movement, and tissue deformation, while anticipating the next movement of the console surgeon’s instruments to avoid instrument collisions and obstruction of the operative view. Previous studies have shown that 3D visualization on the assistant side can shorten the time required to complete complex tasks [9], indirectly indicating that the assistant’s work entails distinct visuocognitive and technical requirements. The assistant is not the surgeon’s passive “third hand”, but a key team member who helps complete the procedure under constrained conditions.

In summary, robotic surgery training should not end with operator credentialing, nor should individuals with only basic laparoscopic experience automatically be considered qualified robotic surgery assistants. A bedside assistant in robotic surgery requires structured training and assessable competency standards, including safe instrument insertion, precise suction and exposure, dynamic retraction, vascular clip application, use of linear cutting staplers, management of unexpected bleeding, and emergency conversion. A double-surgeon training model is feasible for improving assistants’ laparoscopic proficiency in robotic surgery [10]. As robotic staplers, integrated suction systems, and new platforms continue to evolve, the division of tasks between the console surgeon and the assistant may change; nevertheless, adequate exposure, precise hemostasis, low-tension reconstruction, and emergency management remain constant requirements of surgery. Robotic technology has changed the console surgeon’s operative interface, but it has not rendered team-wide laparoscopic competence unnecessary. The future of minimally invasive surgery requires not only skilled console surgeons, but also highly competent bedside assistants who have undergone systematic training and can exercise independent judgment and operate safely.
